# Highlights on Genomics Applications for Lysosomal Storage Diseases

**DOI:** 10.3390/cells9081902

**Published:** 2020-08-14

**Authors:** Valentina La Cognata, Maria Guarnaccia, Agata Polizzi, Martino Ruggieri, Sebastiano Cavallaro

**Affiliations:** 1 Institute for Biomedical Research and Innovation, National Research Council, Via P. Gaifami 18, 95126 Catania, Italy; valentina.lacognata@cnr.it (V.L.C.); maria.guarnaccia@cnr.it (M.G.); 2 Chair of Pediatrics, Department of Educational Sciences, University of Catania, Via Casa Nutrizione, 39, 95124 Catania, Italy; agata.polizzi1@unict.it; 3 Unit of Rare Diseases of the Nervous System in Childhood, Department of Clinical and Experimental Medicine, Section of Pediatrics and Child Neuropsychiatry, AOU “Policlinico”, PO “G. Rodolico”, Via S. Sofia, 78, 95123 Catania, Italy; m.ruggieri@unict.it

**Keywords:** lysosomal storage diseases, diagnosis, genomics, newborn screening

## Abstract

Lysosomal storage diseases (LSDs) are a heterogeneous group of rare multisystem genetic disorders occurring mostly in infancy and childhood, characterized by a gradual accumulation of non-degraded substrates inside the lysosome. Although the cellular pathogenesis of LSDs is complex and still not fully understood, the approval of disease-specific therapies and the rapid emergence of novel diagnostic methods led to the implementation of extensive national newborn screening (NBS) programs in several countries. In the near future, this will help the development of standardized workflows aimed to more timely diagnose these conditions. Hereby, we report an overview of LSD diagnostic process and treatment strategies, provide an update on the worldwide NBS programs, and discuss the opportunities and challenges arising from genomics applications in screening, diagnosis, and research.

## 1. Introduction

Lysosomal storage diseases (LSDs) are a heterogeneous group of heritable (inborn) metabolism defects that affect the function of lysosomes. This group comprises about 70 monogenic disorders of lysosomal catabolism and is characterized by a gradual accumulation of non-degraded substrates inside the lysosome, which in turn leads to cellular dysfunction, tissue damage, and death [1]. The majority of LSDs are inherited as autosomal recessive traits (except for Fabry, Hunter, and Danon diseases that are X- linked) and are caused by mutations in genes encoding lysosomal proteins (i.e., acidic hydrolases, integral membrane proteins, and activator or carrier proteins) whose functional deficiencies trigger the pathogenetic cascade [2]. Although each disorder is rare, per se, with estimated incidences ranging from 1 in 50,000 to 1 in 250,000 live births, LSDs as a group are relatively common disorders (1:5000 live births) [1].

**Figure 1:**
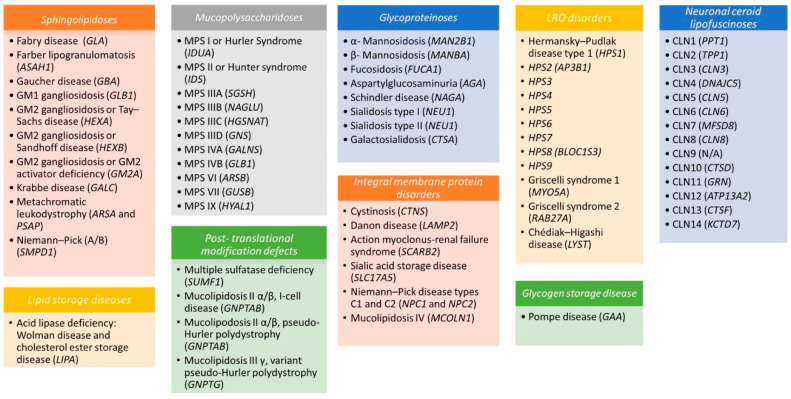
Disorders of lysosomes and lysosome-related organelles (LROs). Lysosomal storage diseases (LSDs) and LROs have been subclassified according to the biochemical type of stored material (sphingolipidoses, mucopolysaccharidoses, glycoproteinoses, lipid storage diseases) or to the integral membrane proteins, post-translational modification, and lipofuscin metabolism defects. The causative gene is specified in parentheses.

LSDs can be categorized by (a) the biochemical type of stored material (e.g., sphingolipidoses, mucopolysaccharidoses, glycoproteinoses); (b) the post-translational modification of lipofuscin degradation or metabolism defects; (c) the dysfunctions in membrane proteins; or (d) the altered lysosome-related organelle (LRO) defects (Figure 1).

Clinically, LSDs may present with a broad range of phenotypes reflecting the age of onset (e.g., more severe infantile forms vs. later/adult milder forms) [3], the extent and severity of nervous system and/or systemic involvements, and the related variability of signs and/or symptoms. In general, the disease progresses and evolves relentlessly over time. Signs and/or symptoms mainly include facial dimorphisms, psychomotor developmental delay and cognitive decline, seizures, impairment of vision, recurrent infections, muscle deficits, organomegaly, immune defects, and skeletal changes, all having a severe impact on prognosis and influencing the quality of life for patients and families [4,5].

## 2. The Biology of Lysosomes

Lysosomes are the key cellular organelles in macromolecule catabolism, responsible for the breakdown and recycling of a wide range of complex metabolites including glycosides, lipids, phospholipids, proteins, and nucleic acids. This catabolic function is orchestrated by approximately 60 unique acidic hydrolase enzymes (glycosidases, sulfatases, peptidases, phosphatases, lipases, and nucleases), which are located within the lysosomal lumen where the enzyme and the substrate interact with each other. While the enzymes are mainly synthesized within the endoplasmic reticulum (ER), tagged with a mannose-6-phosphate (M6P) residue in the Golgi apparatus and then trafficked into the lysosome [6,7], the substrates are transported through different routes according to the nature of the cargo. Materials from outside the cell are delivered through the endocytosis pathway via clathrin-mediated or caveolin-mediated endocytosis vesicles. The cell’s own macromolecules and metabolites are processed and degraded through autophagy, in one of its functional forms including macroautophagy, microautophagy, and chaperone-mediated autophagy [8]. Recent evidence shows that lysosomes are not only catabolic organelles, but they also function as metabolic hubs controlling nutrient sensing, amino-acid and ion homeostasis, vesicle trafficking, and cellular growth, and they establish contact sites with other organelles (e.g., mitochondria, ER, or peroxisomes) [7,9].

## 3. Diagnosis and Therapeutic Strategies

Diagnosis of LSDs is often a challenge for clinicians, owing to the rarity of single disorders and to the non-specificity of signs and/or symptoms, often attributed to other neurological and/or systemic diseases. Before the spread of expanded newborn screening, individuals were diagnosed years after sign and/or symptom onset, when the disease was already advanced and interventions proved less efficacious [10].

**Figure 2:**
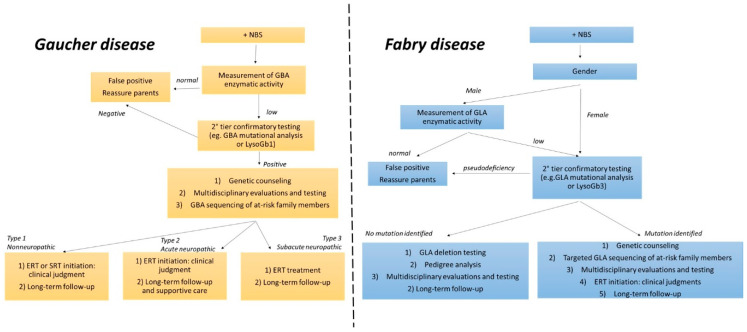
Diagnostic flowchart for Gaucher and Fabry diseases. NBS, newborn screening; LysoGb1, glucosylphingosine; LysoGb3, globotriaosylphingosine; ERT, enzyme replacement therapy; SRT, substrate reduction therapy.

The typical diagnostic work-up for LSDs includes taking a detailed history on the clinical presentation(s) and course of disease; measuring the activity of single enzymes or protein abundance in leukocytes, fibroblasts, urine, or rehydrated dried blood spots (DBS); conducting various laboratory, ophthalmological, otolaryngology, ultrasonographic, neurophysiological, and imaging investigations [11]. When enzyme levels fall below the average, second-tier confirmatory biomarker tests and gene sequencing are performed to identify DNA-specific mutations affecting gene function (Figure 2) [2]. Moreover, prenatal diagnosis for some LSDs is emerging, using uncultured chorionic villi [12,13].

**Figure 3:**
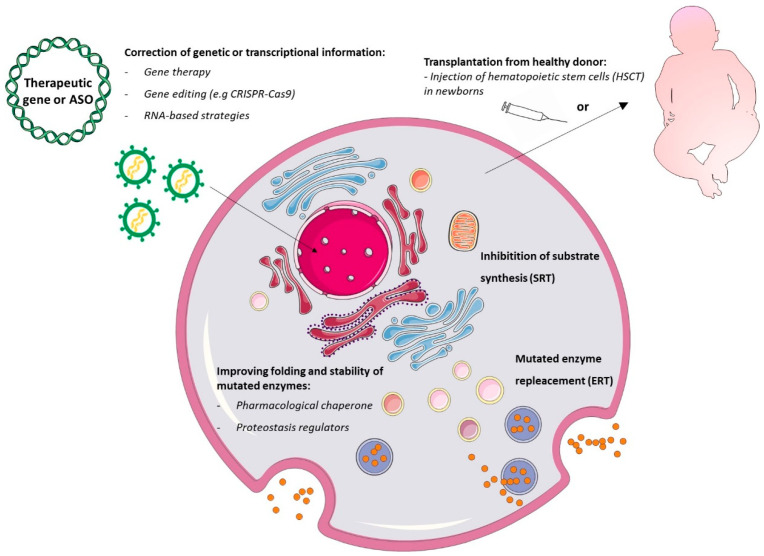
Current lysosomal storage disorder therapeutic strategies. Gene therapy and gene editing approaches aim to introduce a functional gene or to correct the defective gene or transcript inside cells. Hematopoietic stem cell transplantation (HSCT) repopulates specific tissues and allows local release of functional lysosomal hydrolases by healthy donor cells. Substrate reduction therapy (SRT) inhibits substrate synthase at the early Golgi compartment. Enzyme replacement therapies (ERTs) deliver functional enzymes to lysosomes via the endocytosis pathway. Chaperones can stabilize the mutant enzyme and partially restore catalytic activity in the endoplasmic reticulum (ER) and overall in lysosomes. Lastly, proteostasis modifiers stabilize transcription factors within the nucleus, improve lysosomal enzyme expression and translation in the ER, reduce lysosomal membrane permeability, and improve lysosomal function.

Major advances have been made in recent years in our understanding of the pathophysiology of LSDs [1]. This has allowed researchers to identify multiple potential clinical interventions, targeting different events in the pathogenetic cascade (Figure 3), and enabled clinicians to expand, at least for some LSDs, the opportunities for therapeutic strategies besides the supportive medical and physical therapies (e.g., management of neurological complications, ventilatory or nutritional support, orthopedic interventions in order to alleviate deformities, and several others) [14].

The cornerstone for current treatment paradigms is enzyme replacement therapy (ERT), which is considered the standard of care for Gaucher, Fabry, Pompe disease, and mucopolysaccharidosis I (MPSI) [15]. Approved around the nineties by the US Food and Drug administration (FDA) Committee for ERT, the current recombinant functional enzyme is delivered via periodic intravenous infusion whose uptake occurs via the endocytosis pathway. Timely initiation of treatment is crucial for an optimal clinical outcome [16]. Major disadvantages of ERT still include the inability of the recombinant enzymes to diffuse easily in all affected tissues (in particular into the central nervous system, due to their large size), [17], the great variability in patient immune responses, and the transient therapeutic benefit [15].

Hematopoietic stem cell transplantation (HSCT) uses hematopoietic stem cells from healthy donors. These cells can repopulate specific tissues and locally release functional lysosomal hydrolases in the extracellular space and into the blood circulation [18]. Despite the high morbidity and mortality rate due to graft rejection and infection, this therapy remains the first choice of treatment for children with MPS IH (Hurler disease), since it prevents the development of neurological symptoms and increases life expectancy, especially when performed early [19].

Other strategies are directed towards restoring the equilibrium between substrate synthesis and degradation by lysosomal enzymes (the storage equation of LSDs) [7]. In particular, substrate reduction therapy (SRT) approved by the FDA in 2003 (e.g., miglustat, employed for Gaucher disease) uses small-molecule enzyme inhibitors that slow down the build-up process of macromolecules, reducing the storage amount in lysosomes, and inhibiting the biosynthetic pathways [20]. In contrast to ERT, SRT drugs are orally administered and are stable at ambient temperature. They do not generate immune reactions and cross the blood–brain barrier, but they have a slower onset of action than ERT and produce adverse effects or complications relating to drug metabolism [7].

Pharmacological chaperone therapy (PCT) (e.g., migalastat for Fabry disease) uses inhibitory molecules, which target mutant lysosomal enzymes to favor their native conformational folding, stability, catalytic activity, and correct trafficking, also extending their half-life [8]. Some studies evidenced that migalastat provides clinical results comparable to those of ERT [21]. Chaperons were also used efficaciously in combination with ERT to treat patients with Pompe disease [22]. Unfortunately, this kind of treatment is mutation-sensitive and clinically impractical owing to the great efforts needed to find an optimal drug dosage.

New-generation pharmacological strategies are rapidly advancing into clinical trials, including proteostasis modifiers and gene therapy.

Proteostasis modifiers are able to regulate the components of the proteostasis machinery (a multiple regulatory integrated system including protein synthesis, structural folding, post-translational modification, trafficking, and degradation) and have already been suggested for treating Gaucher disease and GM2 gangliosidosis (Tay–Sachs disease) [8].

LSDs are excellent candidates for gene therapy for many reasons: not only are they well-known single-gene disorders, but also the expression of the enzyme is generally not subject to complex regulatory mechanisms. In addition, even a small increase in enzymatic activity is sufficient to revert the clinical phenotype [23]. A broad range of different strategies may be exploited depending on the tissues that need to be targeted and the characteristics of the protein that must be replaced. Expression cassettes containing the functional gene may be delivered to cells via direct gene transfer (by viral-based systems) or via indirect gene transfer (by re-implanting engineered autologous patient stem cells back into the donor) [24].

Promising perspectives come from genome editing platforms (ZFN, TALEN, and CRISPR-Cas9 systems), which have recently enabled the possibility of modifying target sites within the genome in a precise manner [25,26]. The combination of nuclease-mediated genome editing with autologous hematopoietic stem cells or induced pluripotent stem cells (iPSCs) may represent a milestone for treatment of LSDs, as it would lower the overall risk of infection during treatment and avoid rejection (graft-versus-host disease) [25,26]. Both preclinical (in vitro, in vivo, and ex vivo) and clinical studies using different editing-based strategies have already been started including trials for mucopolysaccharidoses [27] and GM2-gangliosidoses [28]. The opportunities granted by RNA-based therapies are equally interesting, as they support the feasibility of reverting the LSD phenotype by partially rescuing splicing defects [29]. Indeed, about 5–19% of LSD-causing mutations affect the pre-mRNA splicing process, and in some LSDs, single splicing changes can account for up to 40–70% of pathogenic alleles [29]. Two main splicing therapy strategies have been used for LSDs: (i) modified U1 small nuclear RNAs (U1snRNA) tested in cellular models of Sanfilippo C disease (or Mucopolysaccharidosis IIIC) [30] and Fabry disease [31], and (ii) the antisense oligonucleotide (AONs) approach used in cellular models of Niemann Pick C [32] and for the late-onset form of Pompe disease [33,34].

## 4. LSD Worldwide Newborn Screenings and Methodological Approaches

The rationale for mandatory (expanded) NBSs has historically included serious health conditions with an effective therapy, relatively easy and reliable disease markers, and evidence for the beneficial effect of early treatment in preventing severe disabilities or even death (Wilson and Jungner screening criteria). However, many adaptations on Wilson and Jungner criteria have occurred in the last decades, reflecting better knowledge of the natural history of many such conditions, new appraisals on logistical and ethical issues, and the advances in genetic technology [35,36].

**Figure 4:**
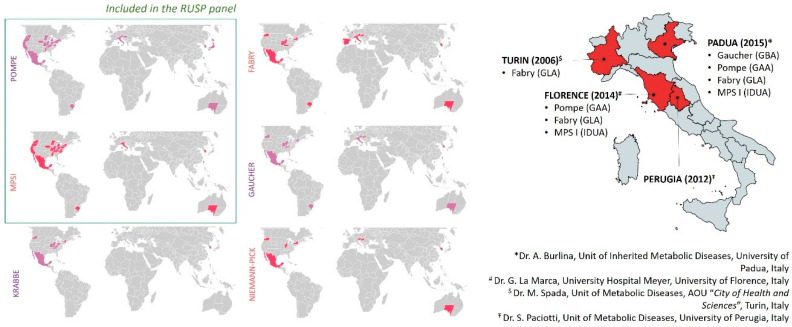
Newborn screening programs (NBSs). The left side shows the worldwide distribution of NBSs including Pompe, MPSI, Krabbe, Fabry, Gaucher, and Niemann Pick diseases. Screening programs are mandated for some regions, under development, or in pilot phases for others (see Supplementary Table S1). The right side shows the pilot Italian regional screening programs for LSDs (brackets indicate the targeted genes; please refer to references reported above).

Until 2006, when the FDA approved alfa-glucosidase for the treatment of Pompe disease [1], none of the LSDs was included in NBS programs. The concept of using DBS extracts for lysosomal enzyme testing, as well as the existence of therapeutic options and the development of new screening tests, opened up the potential for NBS in LSDs. Although widespread internationally, the inclusion of LSDs in expanded NBS is still debated as reflected by the large differences in screening programs worldwide (Figure 4 and Supplementary Table S1). The most active country is certainly the US, where the Advisory Committee on Heritable Disorders in Newborns and Children (ACHDNC) was charged to draw up national recommendations for guiding and supporting states in the development of screening programs. The ACHDNC set out the Recommended Uniform Screening Panel (or RUSP), a list of diseases including 35 core conditions and 26 secondary conditions, the Committee recommends every baby should be screened for. Among LSDs, Pompe disease and MPS I are considered the most favored for inclusion, and, since 2016, these two conditions have been included in RUSP (Figure 4) [37,38]. Meanwhile, pilot LSD screening programs have been implemented in a number of countries worldwide, including Italy [39,40]. In Northern and Central Italy, in particular, four regions (Piemonte, Veneto, Tuscany, and Umbria) started expanded NBS programs for Pompe, Fabry, Gaucher, and MPSI promoted by regional Health Government indications (Figure 4), foreshadowing its extension in Italy to a larger newborn population [41,42,43,44,45,46,47]. As a consequence, thanks to an amendment published by the Italian government in December 2018 (Gazzetta Ufficiale n. 302), LSDs are currently included in the national screening program.

The two main platforms currently used for screening multiple LSDs are the digital microfluidic fluorometry (DMF) platform and the tandem mass spectrometry (MS/MS) platform [37,48,49,50,51]. DMF is the multiplexing advancement of the classical enzymatic fluorescent assay based on synthetic 4-methylumbelliferone (4-MU). It has been developed by Advanced Liquid Logic, Inc. (now Baebies, Inc. Durham, NC, USA) and is currently used for clinical diagnostic purposes and for NBSs. DMF is based on submicroliter droplets, which are moved on an electrode-plate chip through a process known as electrowetting. In this particular “spatial multiplexing”, each LSD enzyme reaction is performed on a single droplet under its individually optimized conditions (pH, inhibitors, buffer). The latest version simultaneously measures the activity of five enzymes to diagnose MPS-I, MPS-II, Pompe, Fabry, and Gaucher diseases. MS/MS is used to detect in a 6-plex assay the enzymatic products responsible for Pompe, MPS-I, Fabry, Gaucher, Krabbe, and Niemann-Pick-A/B diseases. The method currently commercialized as NeoLSD by PerkinElmer Corp starts with an incubation phase of the sample with a mixture of the six substrates and internal standards in a single buffer, followed by liquid–liquid extraction and flow-injection MS/MS. Detailed comparative information (space and manpower requirements, approximate costs, analytic precision) between MS/MS vs. DMF assays are listed in previous studies [37,48,49,51]. The DMF platform workflow is simpler, requires less maintenance, and generates results faster than MS/MS, providing results within the same day of specimen analysis. Conversely, MS/MS is more accurate and precise, and it can be used to assay biomarkers for which no fluorimetric methods exist [37,48,49,51].

## 5. Opportunities and Challenges for Genomics in LSDs

**Figure 5:**
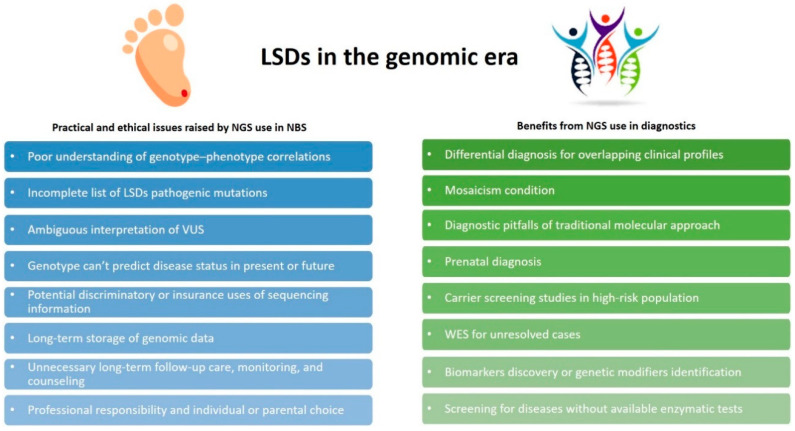
Next generation sequencing (NGS) applications in LSDs. Practical and ethical issues raised by the use of NGS in NBS are shown in the blue boxes; benefits derived from NGS use in diagnostic settings are shown in the green boxes.

Genome-scale sequencing provides a powerful diagnostic tool for patients affected by conditions, which escape the diagnosis by traditional genetic investigation, and offers a range of new opportunities for genomic medicine. With the overwhelming entry of massive parallel sequencing (NGS) into modern medicine, scientists and clinicians started to wonder whether genomic sequencing could replace conventional biochemical tests by improving the screening of newborns or if it could just be useful as an optional supplementary tool [52]. A major driving force for this alternative approach was the rapidly decreasing price of NGS (competitive with current NBS prices) coupled with the vastly improved read depths and accuracy of sequencing platforms [53]. Considerations about the context of using sequencing information are addressed elsewhere in this issue (Figure 5).

In a practical diagnostic setting, the goal of genome-scale sequencing is to identify genetic variants so as to provide a molecular etiology for patients’ clinical manifestations, considering all other variants as incidental findings. The high-throughput ability of NGS has been successfully used to diagnose LSDs [54,55,56,57,58,59], both in the form of exome and targeted sequencing. This is particularly useful when applied to specific diagnostic contexts, including carrier screening studies in high-risk populations (e.g., the Ashkenazi Jewish population) [60,61], prenatal diagnosis [62], unsolved cases where traditional molecular diagnostic approaches have failed [63], unclear or suspected LSD cases [64,65], as well as in defining genotype–phenotype correlations [66] or to find out genetic disease modifiers [67]. More interesting is the use of NGS to differentiate genetically heterogeneous diseases with overlapping clinical phenotypes, such as Pompe disease, limb-girdle muscular dystrophies [68,69], and Gangliosidosis [70], or to investigate mosaic conditions [71,72]. Many companies developed commercial panels and offer direct-to-consumer sequencing services for suspected LSD cases, utilizing custom panels that target few or many genes (causative genes, lysosomal pathway-related genes, or peroxisome disorder-related genes) and are based on arbitrary research (Supplementary Table S2).

In contrast, the application of genomic sequencing for NBS substantially raises a whole host of issues encompassing several disciplines, which need to be appropriately discussed. One major challenge is how to accurately interpret the clinical significance of incidental findings (variants of unknown significance, VUS), including peri-gene sequence variants, mutations localized in intronic regions and in UTRs, or synonymous variants having an impact on gene regulation. Mutational databases for LSDs are already available; however, the complete list of LSD pathogenic mutations is still expanding. Moreover, there is a lack of sufficiently large ethnicity-specific genetic datasets leading, in turn, to the risk of falling into free interpretation of VUS. A combined international effort to generate large, freely available datasets, better prediction algorithms, and standardized tests in terms of laboratory work (e.g., sequencing platforms, read depths, mean and minimum coverage) should be regarded as of primary importance to solve data interpretation [53]. More importantly, there is still a poor understanding of genotype-phenotype correlations. Most LSD patients are complex heterozygotes, the pathogenicity of many alleles is still unknown, and genotype characterization is not enough to predict disease status in the present or the future [53]. NGS might reveal patients in their early years with adult-onset disease, who might not require treatment for decades, if not at all. Further concerns regard the professional responsibility and individual or parental choices about the types of findings that should be reported, potential discriminatory or insurance uses of sequencing information, long-term storage of genomic data, unnecessary interventions, and costly long-term follow-up care, monitoring, and counseling [52,73].

Nonetheless, attempts to expand the use of DNA sequencing in NBS have been carried out using customized NGS panels targeting few relevant genes or alternatively using a WES approach and bioinformatics analysis ad hoc [74,75]. On the other hand, genotyping-based NBS seems the better solution for high-risk neonates admitted to the neonatal intensive care unit (NICU), or for those disorders with no existing biochemical marker [76,77]. For example, in the case of nephropathic cystinosis, PCR- and NGS-based analyses have been used for NBS [78]. Therefore, as outlined by the Newborn Sequencing in Genomic Medicine and Public Health (NSIGHT) consortium, “sequencing technology can be beneficially used in newborns when its use is nuanced and attentive to context” [73].

## 6. Second-Tier Confirmatory Biomarkers: Which One?

The increasing number of NBS pilot studies worldwide has revealed an unexpectedly high number of false-positive samples and has highlighted the need to introduce second-tier testing in order to reduce the recall rate and assist in disease diagnosis. Several primary or secondary accumulating metabolites (i.e., molecules directly or indirectly enhanced as a result of defective lysosomal function) have been proposed as candidate biomarkers, as they are easily detectable in plasma or urine [3,79]. Lysosphingolipids for sphingolipidoses (LysoGb1 for Gaucher disease, LysoGb3 for Fabry disease, LysoSM and LysoSM509 for Niemann-Pick disease type A/B and C, GalSph for Krabbe disease), heparan and dermatan sulphates for MPSs, and glucose tetrasaccharide for Pompe have been proposed as candidate confirmatory biomarkers for differentiating patients with pathogenic mutations, pseudodeficiency alleles, and/or benign variants at the time of screening [39,80,81,82,83,84,85]. However, uncertainties remain in the use of these metabolic biomarkers, and further studies are necessary to achieve the development of definitive pipelines.

For the past few years, the search for new biomarkers has shifted towards microRNAs [86,87,88], opening an interesting perspective for genomics applications. Indeed, specific patterns of circulating miRNAs can identify patients with Fabry or Pompe diseases, and they could be useful for predicting the evolution of the disease or for assessing responses to therapy as well. Some of these miRNAs, in particular, were associated with heart problems and endothelial dysfunctions in Fabry patients, or they significantly correlated with phenotype severity, muscle dysfunctions, and Pompe disease-related patho/pathways (autophagy, muscle regeneration, muscle atrophy) [86,87,88].

## 7. The Importance of a Timely Diagnosis

As anticipated in the introduction, LSDs are rare genetic diseases not frequently encountered in the medical practice, and they often receive inadequate clinical and social consideration compared to other disorders. Nevertheless, taken together, they affect a certain percentage of the overall population (i.e., 1:5000 live births) mostly in infancy or childhood, although patients with late-onset/milder phenotypes are expected and represent the most subtle and difficult cases to identify. Indeed, while positive-NBS children with a pediatric onset of LSDs are followed by expert pediatricians, late-onset patients without a previous clinical history may be misdiagnosed for many years. Unfortunately, no pathognomonic traits for LSDs exist, as they are characterized by a non-specific, likely equivocal multisystemic symptomatology that combines systemic manifestations with overlapping neurological signs, and they are often indistinguishable even biochemically (e.g., MPSIII/Sanfilippo syndromes A-B-C-D, Table 1). These clinical and practical issues aggravate the quality of life for patients and families, as they not only suffer from their disease manifestations but also undergo continuous psychological stress caused by the uncertainty of both diagnostic responses, uncertain outcomes, and non-resolutive therapeutic solutions.

Nowadays, genetic analysis represents the only valid aid to rapidly diagnose and differentiate suspected LSDs cases, allowing causative mutations to be identified. The need to introduce broad and ad hoc designed genetic tests (e.g., targeted gene panels) in diagnostic workflows is becoming increasingly clear in order to easily identify LSDs and draw up informative guidelines that can properly direct both clinicians and geneticists towards the right criteria for results interpretation and diagnostic report writing. Timely diagnosis through genetic/genomic applications is key to halt disease progression, reduce psychological burden, optimize clinical management, and provide appropriate genetic counseling. Moreover, molecular profiling and genomic sequencing information may prompt the design of novel therapeutic drugs targeting specific mutations. The development of more effective treatments will open the possibility for new clinical trials that address stratified patient subclasses and will pave the way to personalized medicine. This, in the near future, will improve the quality of life of patients and their families and reduce both direct and indirect (e.g., care-givers services) costs to national health services and families.

## 8. Conclusions

LSDs are multisystem disorders with heterogeneous genetic profiles and overlapping clinical manifestations. Although they are monogenic diseases, the procedures and/or protocols for early diagnosis, management, and implementation of newborn screening programs (NBSs) deserve greater attention from the scientific community. Many efforts and elaboration phases by institutional networks and researcher partnerships are ongoing, purposing the production of benefits for patients with these rare, life-threatening diseases. MetabERN (Metabolic European Reference Networkgene, available at https://metab.ern-net.eu/), for example, is a European initiative aimed at developing a real-time consultation platform for clinical decision-making processes, and it fosters translational research programmers across inherited metabolic disease. IMI (Innovative Medicine Initiative, an EU public–private partnership), instead, is a funding project for Horizon 2020 with the goal of shortening the path to diagnosis by using newborn/pediatric genetic screening via application of advanced digital technologies (Call name H2020-JTI-IMI2-2020-23-two-stage). Application of molecular NGS-based testing (either in the form of WES or targeted gene panels) in this perspective represents a real and valuable aid to provide timely and correct diagnosis, detect carriership status, and ensure genetic counseling for family planning, thus improving the overall standards of care for patients and families. Consolidation of existing fragmented efforts will result in improved clinical and patient-oriented outcomes, increase public understanding around rare diseases, and potentially lead to better rare disease policies as well as improved value-based healthcare.

## Figures and Tables

**Table 1:** Disease name, causative genes, age of onset, accumulating substrates, and main clinical manifestations of MPS.

				Head						Chest & Abdomen				Skeletal			Neurologic					
Disease name	Gene	Stored material	Onset of symptoms	Coarse facial features	Hearing loss	Teeth abnormalities	Retinal degeneration	Corneal clouding	Enlarged tongue	Heart valvular disease	Respiratory tract infections	Hernias	Hepatosplenomegaly	Short stature	Joint stiffness and contractures	Dysostosis multiplex	Developmental delay	Cognitive deficits	Neurodegeneration	Sleep disturbances	Hydrocephalus	Seizures
MPS IH	*IDUA*	DS, HS	1 year	+	+	+	+	+	+	+	+	+	+	+	+	+	+	+	+			
MPS IH/S			3–8 years					+		+	+	+	+	+	+	+						
MPS IS			>5 years				+	+		+						+						
MPS II	*IDS*	DS, HS	2–4 years	+	+	+	+		+	+		+	+	+		+		+	+			+
MPS IIIA	*SGSH*	HS	2–6 years	+	+						+		+		+	+	+	+		+		+
MPS IIIB	*NAGLU*			+	+						+		+		+	+	+	+		+		+
MPS IIIC	*HGSNAT*			+	+						+	+	+		+	+	+	+		+		+
MPS IIID	*GNS*			+	+						+		+		+	+	+	+		+		+
MPS IVA	*GALNS*	KS	1–3 years	+	+	+		+		+	+	+	+	+								
MPS IVB	*GLB1*			+	+	+		+		+	+	+	+	+								
MPS VI	*ARSB*	DS	Infancy	+	+			+		+		+	+	+	+	+						
MPS VII	*GUSB*	DS, HS	1–4 years	+	+	+		+		+	+	+	+	+	+	+		+	+		+	
MPS IX	*HYAL1*	HA	<1 year											+	+							

HS: heparin sulfate; DS: dermatan sulfate; KS: kermatan sulfate; HA: hyaluronate.

## Supplementary Materials

The following are available online at https://www.mdpi.com/2073-4409/9/8/1902/s1, Table S1: LSDs newborn screening programs worldwide, Table S2: NGS panels for LSDs diagnosis.
