# Supplementary material for: Highlights on Genomics Applications for Lysosomal Storage Diseases
Source: Cells. 2020 Aug 14;9(8):1902. doi: 10.3390/cells9081902 (PMC7465195; doi:10.3390/cells9081902)
Supplement: Supplementary file 1 [file cells-09-01902-s001.zip › cells-884166-S2 pub.pdf]

NGS panels for LSDs diagnosis

PROVIDER	PANEL NAME	TIME	N. of genes	LINK	COUNTRY
Greenwood Genetic center	Lysosomal Storage Disease Sequencing Panel	8-10 weeks	**75**	www.ggc.org/test-finder-item/lysosomal-storage-disease-74-gene-sequencing-panel	South Carolina, USA
CENTOPEDIA - CENTOGENE	Lysosomal storage disease NGS Panel + CNV	25 busines s days	**50**	www.centogene.com/science/centopedia/lysosomal-storage-diseases-lsd-panel.html	Rostock, Germany and Cambridge, Massachusetts, USA
BiooScientific (a Perkin Elmer company)	NEXTFLEX® Lysosomal Storage Disorders Amplicon Panel	N/A	**11**	www.rafer.es/sites/default/files/enfermedades_deposito_lisomal.pdf	Burleson Rd, Austin, TX, USA
PALM	London Health Sciences Centre Lysosomal Storage Panel (LHSC LSD)	4 – 6 weeks	**50**	www.lhsc.on.ca/palm/molecular/panels.html#storage	London, UK
Eurofins Clinical Diagnostics	Lysosomal Storage Disorders Panel: Sequencing and CNV Analysis	6 weeks	**60**	www.egl-eurofins.com/tests/MM120	Tucker, Georgia, USA
GeneDx	Combined Lysosomal and Peroxisomal Disorders Panel	4 weeks	**82**	www.genedx.com/test-catalog/disorders/lysosomal-storage-disorders/	Gaithersburg, Maryland, USA
	Lysosomal Disorder Panel		**57**		
Asper biogene	Lysosomal Storage Disease NGS panel	6-9 weeks	**50**	www.asperbio.com/asper-metabolic-disorders/lysosomal-storage-disease-ngs-panel/	Tartu, ESTONIA
Invitae Corporation	Invitae Comprehensive Lysosomal Storage Disorders Panel	10–21 calendar days	**48**	www.invitae.com/en/physician/tests/06170/#info-panel-resources	San Francisco, CA, USA
Blue Print Genetics	Lysosomal Disorders and Mucopolysaccharidosis Panel	4 weeks	**102**	blueprintgenetics.com/tests/panels/metabolic-disorders/lysosomal-disorders-and-mucopolysaccharidosis-panel/	Keilaranta Espoo Finland
Mayo Clinic Laboratories	Lysosomal Next-Generation Sequencing	4-5 weeks	**58**	www.mayocliniclabs.com/test-catalog/Clinical+and+Interpretive/605162	Rochester (MN), Phoenix (AZ), Jacksonville (FL) - USA
Fulgent genetics	Lysosomal Disorders NGS Panel	3 - 5 weeks	**106**	www.fulgentgenetics.com/Lysosomal-Disorders	Pavia, Italy
