# Supplementary material for: Highlights on Genomics Applications for Lysosomal Storage Diseases
Source: Cells. 2020 Aug 14;9(8):1902. doi: 10.3390/cells9081902 (PMC7465195; doi:10.3390/cells9081902)
Supplement: Supplementary file 1 [file cells-09-01902-s001.zip › cells-884166-S1 pub.pdf]

LSDs newborn screening programs worldwide

	Pompe	MPSI	Krabbe	Fabry	Gaucher	Niemann-Pick (A/B)	Other MPSs
AMERICA							
California	[1]	*					
Delaware	*	*					
District of Columbia	*	*					
Illinois	[2, 3]	[2]	*	[2, 3]	[2, 3]	[2]	
Kentucky	[4]	[4]	[4]				
Maryland	*	*		*			
Massachusetts	*	*					
Mexico	[5]	[5]	[5]	[5]	[5]	[5]	
Michigan	*	*					
Minnesota	*	*					
Mississippi	*	*					
Missouri	[6]	[6]	*	[6]	[6]		
Nebraska	*	*					
New York	[7]	[7]	[8]	[7]	[7]	[7]	
North Carolina		[9]					
Ohio	*	*	*				
Oregon	*	*					
Pennsylvania	*	*					
Rhode island	*	*					
Tennessee	*	*	*	*	*		
Vermont	*	*					
Virginia	*	*					
Washington	[10, 11]	[10, 11]	[10]	[10, 11]	[10]	[10]	[12]
Rio Grande do Sul (Brazil)	[13]	[13]		[13]	[13]		[14]
EUROPE							
Austria	[15]			[15]	[15]	[15]	
Belgium	[16]	[16]		[16]			
Hungary	[17]			[17]	[17]	[17]	
Italy	[18]	[18, 19]		[18, 20]	[18, 21]		
Spain				[22]			
ASIA							
Japan	[23]						
South Korea	[24, 25]	[24]	[24]	[24, 25]	[24, 25]	[24]	
Shanghai (China)					[26]		
Taiwan	[27-29]	[28]		[28]	[28]		[30]
OCEANIA							
South Australia	[31]	[31]		[31]	[31]	[31]	[31]

* Where otherwise specified, information were extracted from http://babysfirsttest.org/ - update May 2020. Please, refer to this database for updated news.

1. Tang, H.; Feuchtbaum, L.; Sciortino, S.; Matteson, J.; Mathur, D.; Bishop, T.; Olney, R. S., The First Year Experience of Newborn Screening for Pompe Disease in California. *International journal of neonatal screening* **2020,** *6* (1), 9.
2. Burton, B. K.; Charrow, J.; Hoganson, G. E.; Waggoner, D.; Tinkle, B.; Braddock, S. R.; Schneider, M.; Grange, D. K.; Nash, C.; Shryock, H., et al., Newborn Screening for Lysosomal Storage Disorders in Illinois: The Initial 15-Month Experience. *The Journal of pediatrics* **2017,** *190*, 130-135.
3. Burton, B.; Charrow, J.; Angle, B.; Widera, S.; Waggoner, D., A Pilot Newborn Screening Program for Lysosomal Storage Disorders (LSD) in Illinois. *Molecular genetics and metabolism* **2012,** *105* (2), S23-S24.
4. Minter Baerg, M. M.; Stoway, S. D.; Hart, J.; Mott, L.; Peck, D. S.; Nett, S. L.; Eckerman, J. S.; Lacey, J. M.; Turgeon, C. T.; Gavrilov, D., et al., Precision newborn screening for lysosomal disorders. *Genetics in Medicine* **2017,** *20* (8), 847-854.
5. Navarrete-Martínez, J. I.; Limón-Rojas, A. E.; Gaytán-García, M. d. J.; Reyna-Figueroa, J.; Wakida-Kusunoki, G.; Delgado-Calvillo, M. d. R.; Cantú-Reyna, C.; Cruz-Camino, H.; Cervantes-Barragán, D. E., Newborn screening for six lysosomal storage disorders in a cohort of Mexican patients: Three-year findings from a screening program in a closed Mexican health system. *Molecular genetics and metabolism* **2017,** *121* (1), 16-21.
6. Hopkins, P. V.; Campbell, C.; Klug, T.; Rogers, S.; Raburn-Miller, J.; Kiesling, J., Lysosomal Storage Disorder Screening Implementation: Findings from the First Six Months of Full Population Pilot Testing in Missouri. *The Journal of pediatrics* **2015,** *166* (1), 172-177.
7. Wasserstein, M. P.; Caggana, M.; Bailey, S. M.; Desnick, R. J.; Edelmann, L.; Estrella, L.; Holzman, I.; Kelly, N. R.; Kornreich, R.; Kupchik, S. G., et al., The New York pilot newborn screening program for lysosomal storage diseases: Report of the First 65,000 Infants. *Genetics in Medicine* **2018,** *21* (3), 631-640.
8. Orsini, J. J.; Kay, D. M.; Saavedra-Matiz, C. A.; Wenger, D. A.; Duffner, P. K.; Erbe, R. W.; Biski, C.; Martin, M.; Krein, L. M.; Nichols, M., et al., Newborn screening for Krabbe disease in New York State: the first eight years' experience. *Genetics in Medicine* **2016,** *18* (3), 239-248.
9. Taylor, J. L.; Clinard, K.; Powell, C. M.; Rehder, C.; Young, S. P.; Bali, D.; Beckloff, S. E.; Gehtland, L. M.; Kemper, A. R.; Lee, S., et al., The North Carolina Experience with Mucopolysaccharidosis Type I Newborn Screening. *The Journal of pediatrics* **2019,** *211*, 193-200.e2.
10. Elliott, S.; Buroker, N.; Cournoyer, J. J.; Potier, A. M.; Trometer, J. D.; Elbin, C.; Schermer, M. J.; Kantola, J.; Boyce, A.; Turecek, F.; Gelb, M. H.; Scott, C. R., Pilot study of newborn screening for six lysosomal storage diseases using Tandem Mass Spectrometry. *Molecular genetics and metabolism* **2016,** *118* (4), 304-309.
11. Scott, C. R.; Elliott, S.; Buroker, N.; Thomas, L. I.; Keutzer, J.; Glass, M.; Gelb, M. H.; Turecek, F., Identification of Infants at Risk for Developing Fabry, Pompe, or Mucopolysaccharidosis-I from Newborn Blood Spots by Tandem Mass Spectrometry. *The Journal of pediatrics* **2013,** *163* (2), 498-503.
12. Scott, C. R.; Elliott, S.; Hong, X.; Huang, J.-Y.; Kumar, A. B.; Yi, F.; Pendem, N.; Chennamaneni, N. K.; Gelb, M. H., Newborn Screening for Mucopolysaccharidoses: Results of a Pilot Study with 100 000 Dried Blood Spots. *The Journal of pediatrics* **2020,** *216*, 204-207.
13. Camargo Neto, E.; Schulte, J.; Pereira, J.; Bravo, H.; Sampaio-Filho, C.; Giugliani, R., Neonatal screening for four lysosomal storage diseases with a digital microfluidics platform: Initial results in Brazil. *Genetics and molecular biology* **2018,** *41* (2), 414-416.
14. Giugliani, R., Newborn screening for lysosomal diseases: current status and potential interface with population medical genetics in Latin America. *Journal of inherited metabolic disease* **2012,** *35* (5), 871-877.
15. Mechtler, T. P.; Stary, S.; Metz, T. F.; De Jesús, V. R.; Greber-Platzer, S.; Pollak, A.; Herkner, K. R.; Streubel, B.; Kasper, D. C., Neonatal screening for lysosomal storage disorders: feasibility and incidence from a nationwide study in Austria. *The Lancet* **2012,** *379* (9813), 335-341.

16. Eyskens, F.; Devos, S., Newborn Screening for Lysosomal Storage Disorders in Belgium. *Journal of Inborn Errors of Metabolism and Screening* **2017,** *5,* 232640981774423.
17. Wittmann, J.; Karg, E.; Turi, S.; Legnini, E.; Wittmann, G.; Giese, A.-K.; Lukas, J.; Gölnitz, U.; Klingenhäger, M.; Bodamer, O.; Mühl, A.; Rolfs, A., Newborn Screening for Lysosomal Storage Disorders in Hungary. **2012,** *6,* 117-125.
18. Burlina, A. B.; Polo, G.; Salviati, L.; Duro, G.; Zizzo, C.; Dardis, A.; Bembi, B.; Cazzorla, C.; Rubert, L.; Zordan, R.; Desnick, R. J.; Burlina, A. P., Newborn screening for lysosomal storage disorders by tandem mass spectrometry in North East Italy. *Journal of inherited metabolic disease* **2017,** *41* (2), 209-219.
19. Burlina, A.; Gueraldi, D.; Polo, G.; Rubert, L.; Cazzorla, C.; Giuliani, A.; Burlina, A., Newborn screening for MPS I: The clinical benefit. *Molecular genetics and metabolism* **2020,** *129* (2), S35-S36.
20. Spada, M.; Pagliardini, S.; Yasuda, M.; Tukel, T.; Thiagarajan, G.; Sakuraba, H.; Ponzone, A.; Desnick, R. J., High Incidence of Later-Onset Fabry Disease Revealed by Newborn Screening*. *The American Journal of Human Genetics* **2006,** *79* (1), 31-40.
21. Burlina, A.; Polo, G.; Gueraldi, D.; Rubert, L.; Cazzorla, C.; Giuliani, A.; Burlina, A., High incidence of Gaucher disease in northeast Italy: Results from lysosomal newborn screening. *Molecular genetics and metabolism* **2020,** *129* (2), S36.
22. Colon, C.; Ortolano, S.; Melcon-Crespo, C.; Alvarez, J. V.; Lopez-Suarez, O. E.; Couce, M. L.; Fernández-Lorenzo, J. R., Newborn screening for Fabry disease in the north-west of Spain. *European journal of pediatrics* **2017,** *176* (8), 1075-1081.
23. Momosaki, K.; Kido, J.; Yoshida, S.; Sugawara, K.; Miyamoto, T.; Inoue, T.; Okumiya, T.; Matsumoto, S.; Endo, F.; Hirose, S.; Nakamura, K., Newborn screening for Pompe disease in Japan: report and literature review of mutations in the GAA gene in Japanese and Asian patients. *Journal of human genetics* **2019,** *64* (8), 741-755.
24. Han, M.; Jun, S.-H.; Song, S. H.; Park, K. U.; Kim, J. Q.; Song, J., Use of Tandem Mass Spectrometry for Newborn Screening of 6 Lysosomal Storage Disorders in a Korean Population. *The Korean Journal of Laboratory Medicine* **2011,** *31* (4), 250.
25. Cho, S. E.; Kwak, J. R.; Lee, H.; Seo, D. H.; Song, J., Triplex tandem mass spectrometry assays for the screening of 3 lysosomal storage disorders in a Korean population. *Clinica Chimica Acta* **2016,** *454,* 20-27.
26. Kang, L.; Zhan, X.; Gu, X.; Zhang, H., Successful newborn screening for Gaucher disease using fluorometric assay in China. *Journal of human genetics* **2017,** *62* (8), 763-768.
27. Chien, Y. H.; Chiang, S. C.; Zhang, X. K.; Keutzer, J.; Lee, N. C.; Huang, A. C.; Chen, C. A.; Wu, M. H.; Huang, P. H.; Tsai, F. J.; Chen, Y. T.; Hwu, W. L., Early Detection of Pompe Disease by Newborn Screening Is Feasible: Results From the Taiwan Screening Program. *Pediatrics* **2008,** *122* (1), e39-e45.
28. Liao, H.-C.; Chiang, C.-C.; Niu, D.-M.; Wang, C.-H.; Kao, S.-M.; Tsai, F.-J.; Huang, Y.-H.; Liu, H.-C.; Huang, C.-K.; Gao, H.-J., et al., Detecting multiple lysosomal storage diseases by tandem mass spectrometry — A national newborn screening program in Taiwan. *Clinica Chimica Acta* **2014,** *431,* 80-86.
29. Yang, C.-F.; Liu, H.-C.; Hsu, T.-R.; Tsai, F.-C.; Chiang, S.-F.; Chiang, C.-C.; Ho, H.-C.; Lai, C.-J.; Yang, T.-F.; Chuang, S.-Y.; Lin, C.-Y.; Niu, D.-M., A large-scale nationwide newborn screening program for pompe disease in Taiwan: Towards effective diagnosis and treatment. *American Journal of Medical Genetics Part A* **2014,** *164* (1), 54-61.
30. Chien, Y.-H.; Lee, N.-C.; Chen, P.-W.; Yeh, H.-Y.; Gelb, M. H.; Chiu, P.-C.; Chu, S.-Y.; Lee, C.-H.; Lee, A.-R.; Hwu, W.-L., Newborn screening for Morquio disease and other lysosomal storage diseases: results from the 8-plex assay for 70,000 newborns. *Orphanet journal of rare diseases* **2020,** *15* (1),
31. Meikle, P. J.; Grasby, D. J.; Dean, C. J.; Lang, D. L.; Bockmann, M.; Whittle, A. M.; Fietz, M. J.; Simonsen, H.; Fuller, M.; Brooks, D. A.; Hopwood, J. J., Newborn screening for lysosomal storage disorders. *Molecular genetics and metabolism* **2006,** *88* (4), 307-314.
